# Outcomes in Clinical Trials of Inhaled Corticosteroids for Children with Asthma Are Narrowly Focussed on Short Term Disease Activity

**DOI:** 10.1371/journal.pone.0006276

**Published:** 2009-07-17

**Authors:** Ian P. Sinha, Paula R. Williamson, Rosalind L. Smyth

**Affiliations:** 1 Institute of Child Health, University of Liverpool, Liverpool, United Kingdom; 2 Centre for Medical Statistics and Health Evaluation, University of Liverpool, Liverpool, United Kingdom; University of Giessen Lung Center, Germany

## Abstract

**Background:**

Little work has been done to determine which outcomes should be measured in randomised controlled trials (RCTs) in children with asthma. Drug regulatory authorities require that short term disease activity is measured, but other outcome domains are not mandatory for licensing and marketing purposes. We aimed to identify whether any domains were underrepresented in RCTs of regular therapies for children with asthma over a 20 year period, and to examine what consistency there was between RCTs in the outcomes used to assess the domains.

**Methodology/Principal Findings:**

By searching the Cochrane Central Register of Controlled Trials in January 2008, we identified all parallel-group RCTs, published between January 1988 and December 2007, which assessed inhaled corticosteroids (ICS) as regular therapy for children with asthma. We evaluated how frequently RCTs measured the following pre-defined domains: disease activity; disease damage; functional status; quality of life; health resource utilisation; and adverse effects of therapy. Our initial search identified 1668 abstracts, of which 412 were retrieved in full. 159 RCTs, of which 115 involved only children and 44 involved children and adults, were included in the review. Disease activity was measured in 157 RCTs, adverse effects of ICS in 135, functional status in 25, quality of life in 21, and health resource utilisation in 17. No RCT measured long term disease damage, although two used FEV1 as a measure of ‘lung growth’. RCTs were inconsistent in the outcomes used to measure the domains.

**Conclusions:**

Short term disease activity is the most frequently measured outcome domain in RCTs in children with asthma. Effects of regular therapies on functional status, quality of life, and long term consequences of asthma are infrequently assessed. A core set of outcomes, developed using consensus techniques, would standardise the measurement of appropriate outcomes in these RCTs. Involving patients would identify outcomes which are most relevant from their perspective.

## Introduction

Asthma in children is a major global health problem [1], because it is an important cause of morbidity, mortality and economic cost [2], it is the commonest chronic condition in industrialised countries [3], its prevalence is increasing [4], and in many children it is a progressive condition that continues into adulthood [5].

The first line of regular therapy for the control of asthma in children is inhaled corticosteroids (ICS), and recommended additional treatments are long acting beta-2 agonists and leukotriene receptor antagonists [6]. Randomised controlled trials (RCTs) are the most scientifically rigorous method for evaluating the efficacy and safety of these medications [7], but it can be difficult to select the most appropriate outcomes to measure in these studies, because asthma impacts on many aspects of the lives of children. For example, the effects of treatment could include improvement of daily symptoms, quality of life, or physiological tests of lung function such as Forced Expiratory Volume in 1 second (FEV1) or Peak Expiratory Flow Rate (PEFR) [8].

We previously published a systematic review, of studies that determined which outcomes should be measured in clinical trials in children [9]. In this review, we proposed that outcomes measured in RCTs that include children should be considered under six domains: short term measures of disease activity, physical consequence of disease, functional status, family outcomes and quality of life, side effects of therapy and health resource utilisation. We found one study that addressed the outcomes used in clinical trials of regular therapies for childhood asthma, in which the authors ascertained, by questionnaire, the opinions of 14 specialists and researchers about outcomes relating to disease activity, functional status, and quality of life [10]. As this study consulted only a limited group of experts, and did not use recognised consensus techniques, it does not provide a robust basis for recommendations about which domains and outcomes are most appropriate for RCTs of children with asthma.

There have been initiatives to standardise the outcomes which are measured in clinical trials of other conditions. The most notable is the OMERACT collaboration, an international network of clinicians and patients, initially formed in response to the observation that clinical trials of patients with rheumatoid arthritis conducted in the USA measured different outcomes to those conducted in Europe. OMERACT uses structured consensus techniques to determine which outcomes should be measured in clinical trials in a variety of rheumatological conditions [11]–[13]. Initiatives such as these increase the likelihood that all important outcome domains are measured, reduce the measurement of inappropriate outcomes [14], and aid comparison and synthesis of findings between different clinical trials [15], [16]. It has also been suggested that arbitrary or inconsistent outcome selection may lead to clinical trials with unnecessarily large sample sizes [17] and reporting biases [17], [18].

The aim of this systematic review was to assess which outcomes had been measured in clinical trials of ICS in children with chronic asthma between 1988 and 2007, in order to determine whether all relevant domains were represented, and whether there was consistent selection of outcomes within these domains. Secondary objectives were to determine whether the selection of outcome domains has changed between 1988 and 2007, whether domains measured in RCTs exclusively involving children differ from those in studies involving both children and adults, and whether domains measured in publically funded trials differed from those in trials funded by the pharmaceutical industry.

## Methods

### Included studies

In order to ensure that we assessed a group of similar studies, we limited this review to include only RCTs with parallel group design that assessed ICS as a therapy to prevent symptoms or long-term effects of asthma in children. We excluded crossover trials because they are generally of acute interventions, the length of treatment in these studies is typically shorter, and the outcomes they measure may differ from those measured in parallel trials [19]. In order to only include RCTs assessing long term preventative therapy for asthma, we excluded studies with a treatment phase of less than one month. The review was restricted to studies published between January 1988 and December 2007.

### Identification of studies

Using the abbreviated search strategy ‘children AND inhaled corticosteroids AND asthma’, the Cochrane Central Register of Controlled Trials was searched in January 2008. This database comprises RCTs from MEDLINE, and also from conference proceedings and journals not indexed in MEDLINE. The references of identified studies were also screened for other potentially eligible studies. The full search strategy is included in supplementary File S1. One reviewer (IS) assessed trial eligibility, under the supervision of the senior authors (PRW and RLS).

### Data extraction and quality assessment

The same reviewer extracted the following data, and any problems were resolved by discussion with the other two authors:

1. All outcomes measured in the trial

2. If stated, the designated primary outcome, and whether this was described in sufficient detail, including the methods used to measure it, by whom, and when it was measured and analysed.

3. Masking of interventions was examined because the extent to which interventions were masked may affect the choice of outcomes, and whether they were measured objectively or subjectively. The adequacy of masking was categorised as follows.

#### Adequately masked

Authors either clearly describe or imply, in the methods, how the allocated treatment was masked to the patient and family, medical caregiver, and relevant trial personnel involved in measuring outcomes.

#### Inadequately masked

Authors specifically state that the identity of the allocated treatment arm was not masked to at least one of the following: patient and family, medical caregiver, or relevant trial personnel involved in measuring outcomes.

#### Unclear

Unclear from study methods whether masking was adequate or not.

4. Other study features: year of publication; interventions compared; ages of children included; length of study treatment; source of funding; single- or multi-centre

### Data analysis and presentation

For each study that included exclusively children, the data were tabulated and each outcome was grouped into one of the following six outcome domains, some of which were further divided into subdomains [9]: disease activity, physical consequence of disease, functional status, social outcomes and quality of life, side effects of therapy and health resource utilisation. Where it was unclear which domain was appropriate, this was resolved by discussion between the authors.

To assess how the selection of outcomes has changed over time, we divided the period 1988 to 2007 into sixteen separate epochs, each lasting five years. In each epoch we calculated the proportion of studies measuring each outcome domain, and we presented the results as a moving window.

## Results

### Flow of included studies

**Figure 1:**
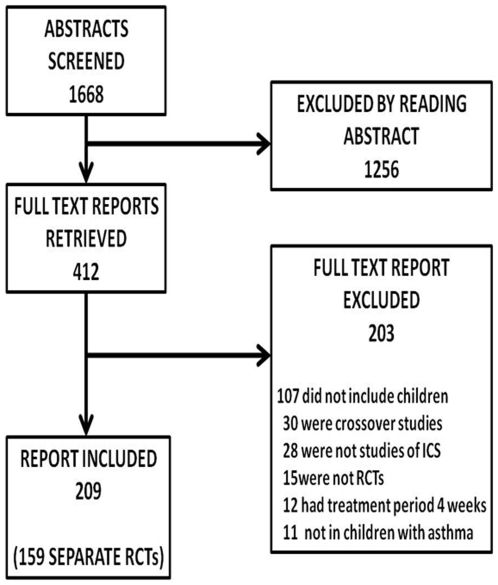
Study flowchart. Flowchart shows the number of abstracts identified by the search, the number of full texts retrieved, and the number of studies included in the review.

The search yielded 1668 potentially eligible reports. Of these, 1256 were excluded by reading the abstract. The remaining 412 were retrieved in full, and 203/412 were subsequently excluded. In total, 209 eligible reports, of 159 RCTs, were included in the review. The review flowchart is shown in Figure 1. Included studies are listed in supplementary File S2.

### Description of included studies

Of the 159 studies included in this review, 115 exclusively included children, and 44 included children and adults.

The characteristics of included studies are summarised in Table 1. Within the group of studies that included only children, all paediatric age groups were represented, but only 25/115 (21%) included children younger than four years of age. In the studies of adults and children, 42/44 (95%) included children aged between 12 and 18 years of age, but not younger than 12, and 2/44 (5%) included children between 5 and 18 years of age, but not younger than 5.

**Table 1:** Characteristics of included studies.

Study characteristic	Category	Number (%) of studies which included only children (n = 115)	Number (%) of studies which included children and adults (n = 44)
**Date of publication**	January 1988 to December 1992	10 (9)	1 (2)
	January 1993 to December 1997	19 (17)	5 (11)
	January 1998 to December 2002	48 (42)	22 (50)
	January 2003 to December 2007	38 (32)	16 (37)
**Length of treatment period**	1 to 3 months	30 (26)	5 (11)
	3 to <6 months	46 (40)	30 (69)
	6 to <12 months	13 (11)	4 (9)
	12 months or longer	24 (21)	5 (11)
	Unclear	2 (2)	0
**Age groups of children included**	<4 years only	17 (15)	0
	<4 and 4 to <12 years	7 (6)	0
	<4 and 4 to<12 and 12 to 18 years	1 (1)	0
	4 to <12 years only	36 (31)	0
	4 to <12 years and 12 to 18 years	53 (46)	2 (5)
	12 to 18 years only	1 (1)	42 (95)
**Number of centres**	Multicentre	75 (65)	42 (95)
	Single centre	39 (34)	2 (5)
	Unclear	1 (1)	0
**Source of funding**	Industry	85 (74)	42 (95)
	Public funding bodies	30 (26)	2 (5)
**Comparisons**	ICS vs Other drug	26 (23)	12 (27)
	ICS vs placebo	25 (22)	4 (9)
	ICS 1 vs ICS 2	14 (12)	3 (7)
	ICS vs same ICS (different delivery device)	12 (10)	2 (5)
	ICS vs same ICS (different dose)	9 (8)	3 (7)
	ICS vs no treatment	1 (1)	0
	ICS 1 vs ICS 2 vs Placebo	16 (14)	10 (23)
	ICS 1 vs ICS 2 vs Other drug	7 (6)	1 (2)
	ICS vs Other drug vs placebo	4 (3)	4 (9)
	ICS vs same ICS (different dose and mode)	1 (1)	0
	ICS 1 vs ICS 1 (different dose) vs ICS 2	0	5 (11)

83/159 (52%) included a comparison between ICS groups (either different doses, modes of delivery, or different types of ICS eg fluticasone vs beclomethasone), 63/159 (40%) included a comparison with placebo, and 54/159 (34%) included a comparison with another drug.

Masking of interventions was deemed adequate in 121/159 studies (76%), inadequate in 33/159 (21%), and unclear in 5/159 (3%). Of the 33 studies that were classed as inadequately masked, 18 compared ICS with another drug, 8 compared ICS administered by different devices, 3 compared one ICS to another, 3 compared ICS administered using different dosing schedules, and 1 compared ICS with no treatment. Subjective outcomes that could have been affected by the lack of blinding were measured in 29/33 of these studies (29/33 measured symptoms, 8/33 measured quality of life and 6/33 measured functional status).

### Outcome domains which were measured in the studies

Disease activity was measured in 157/159 (99%) studies, adverse effects of therapy in 135/159 (85%), functional status in 25/159 (16%), quality of life in 21/159 (13%), and health resource utilisation in 17/159 (11%). No studies measured the effects of ICS on long-term physical consequences of asthma, although two studies measured post-bronchodilator FEV1, as a percentage of the predicted value, to assess ‘lung growth’. In one of these studies children, aged between 5 and 12 years, were randomised to receive inhaled budesonide, nedocromil sodium or placebo for a period of four to six years, and FEV1was measured as the primary outcome [20]. In the other study, patients aged between 5 and 66 years were randomised to treatment with inhaled budesonide or placebo for three years, and FEV1 was measured as a secondary outcome [21]. Similar outcome domains were represented in the 115 studies that included only children and the 44 that included children and adults.

There was a wide variety of outcomes within individual domains. This was greatest for the disease activity domain, which was divided into five subdomains (clinical measures, physiological tests of lung function, global measures, bronchial responsiveness to a challenge agent or exercise and markers of inflammation), each of which included outcomes measured in different ways. As can be seen in Table 2, the selection of subdomains and outcomes was inconsistent across the studies.

**Table 2:** Frequency with which outcome domains, and outcomes used to measure them, were selected in 115 trials involving only children published between 1988 and 2008.

Domain	Subdomain 1	Subdomain 2	Outcome	Number (%) of studies in which measured as primary or secondary outcome n = 115	Number (%) of studies in which measured as primary outcome n = 84^a^
**Disease activity**				**114 (99)**	**74 (88)**
	Clinical measures n = 109	Symptoms	Symptom severity	90 (77)	10 (12)
			Symptom frequency	55 (47)	5 (6)
			Use of rescue therapy	90 (77)	2 (2)
		Exacerbations	Exacerbation frequency	35 (30)	4 (5)
			Time to exacerbation	10 (9)	0
	Tests of lung function n = 103	Spirometry	FEV1	80 (70)	16 (19)
			FVC	31 (26)	0
			Mid expiratory flow	23 (20)	0
			FEV1∶FVC	6 (5)	0
			FEV1 reversibility	9 (8)	0
		PEFR	PEFR	85 (73)	26 (31)
			Diurnal variability	13 (11)	0
			Day-to-day variability	5 (4)	0
		Lung volume	Plethysmographic	4 (3)	0
		Airway flow	Resistance/conductance	5 (4)	2 (2)
	Global measure of control n = 29		Physician-rated	8 (7)	1 (1)
			Parent/patient – rated	14 (12)	0
			‘Treatment failure’	13 (11)	0
			‘Treatment success’	3 (3)	0
	Bronchial responsiveness to a challenge agent n = 29	Induced BHR	Methacholine-induced	26 (22)	6 (7)
			Exercise-induced	7 (6)	0
	Markers of inflammation n = 20		Exhaled nitric oxide	5 (4)	1 (1)
			Leukotriene^b^/interleukin^c^	4 (3)	1 (1)
			Eosinophils^d^/IgE^e^	18 (15)	0
**Physical consequence of disease**				**0**	**0**
**HRU**				**15 (13)**	**1 (1)**
			Unscheduled HRU	15 (13)	1 (1)
**Functional status**				**20 (17)**	**0**
			Effect of asthma on ADL	10 (9)	0
			School attendance	15 (13)	0
**QoL/family outcomes**				**19 (17)**	**0**
			Child's QoL	9 (8)	0
			Caregiver QoL	5 (4)	0
			Caregiver functional status	8 (7)	0
**Adverse Effects of therapy**				**96 (83)**	**14 (16)**
	Routinely monitored AE n = 82		Patient/parent- reported	80 (70)	1 (1)
			Routine laboratory AE	32 (27)	0
			Orophayryngeal infection	28 (24)	0
			Ophthalmological events	7 (6)	0
	H-P-A axis n = 52		Urine/serum cortisol	52 (44)	0
			ACTH stimulation	17 (15)	1 (1)
	Growth n = 41		Growth	41 (35)	9 (11)
			Lower leg growth	1 (1)	1(1)
	Effects of ICS on bone n = 15		Markers of bone turnover	11 (9)	0
			Measures of bone density	8 (7)	2 (2)

Abbreviations used in Table 2: ACTH = adrenocorticotropic hormone; ADL = Activities of Daily Living; AE = adverse events; BHR = bronchial hyperresponsiveness; FEV1 = forced expiratory flow in one second; FVC = forced vital capacity; H-P-A = Hypothalamic-pituitary-adrenal; HRU = health resource utilisation; ICS = inhaled corticosteroids; IgE = Immunoglobulin E; PEFR = peak expiratory flow rate; QoL = Quality of life.

a Of 84 studies that specified primary outcomes, 5 specified co-primary outcomes, and hence the total number of primary outcomes measured is 89.

b Leukotriene LTC4 in serum and nasal secretions and leukotriene LTE4 in urine.

c Interleukins in serum and sputum.

d Eosinophils in serum and sputum, and Eosinophil Cationic Protein in serum and urine.

e IgE in serum.

### Primary outcomes

The primary outcomes measured in the RCTs that included only children are listed in Table 2. It was possible to determine the primary outcome in 84/115 (73%) studies. In 64 of these, the primary outcome was clearly stated by the authors, and in the remaining 20 it was inferred from the outcome used to calculate the sample size. Five studies each selected two co-primary outcomes. Of the 84 studies that specified a primary outcome, 74 (88%) selected primary outcomes that measured disease activity. A total of 17 different primary outcomes were selected, of which physiological measures of airway obstruction, including PEFR (26 studies) and FEV1 (16 studies), were the most frequent. None of the primary outcomes addressed the functional status or quality of life domains

It was possible to determine the primary outcome in 39/44 (89%) studies that included children and adults. In 34 of these, the primary outcome was clearly stated by the authors, and in the remaining 5 it was inferred from the outcome used to calculate the required sample size. In 38/39 (97%), was some measure of disease activity. The most widely used primary outcome was FEV1 (28 studies).

### Outcomes measured in studies funded by the pharmaceutical industry

The frequency with which most domains were measured in the 127 studies sponsored by the pharmaceutical industry was similar to the 32 publicly funded studies. The main difference we observed was that adverse effects of therapy were measured in a higher proportion of studies sponsored by the pharmaceutical industry (118/127, 93%) compared to studies funded from other sources (17/32, 53%).

### How the selection of outcome domains has changed over time

**Figure 2:**
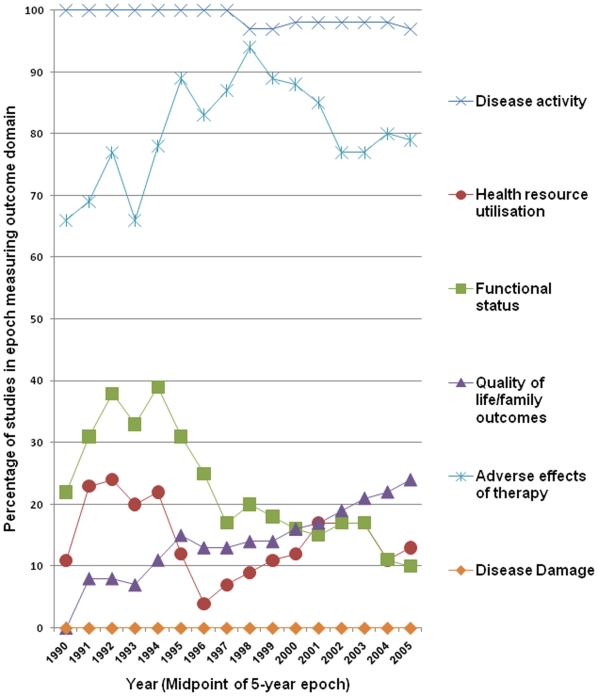
Change in selection of outcomes between 1988 and 2007. The figure shows trends in the measurement of outcome domains in clinical trials of inhaled corticosteroid for children with asthma published between 1988 and 2008. Data are presented as a moving window. Each point along the horizontal axis represents the midpoint of a five year epoch. In each epoch the proportion of studies measuring each individual domain is shown.

The trend over the period January 1988 to December 2007 in the selection of outcome domains in RCTs including only children is shown in Figure 2. Disease activity and adverse effects of therapy have remained consistently frequently measured outcome domains. Since the 1992–1996 epoch the proportion of studies measuring functional status, for example by assessing school absence due to asthma, has decreased from 40% to 10%, and those measuring quality of life have increased from 10% to 25%.

## Discussion

We found that RCTs in children with asthma almost always assess the effects of therapies on short term disease activity, but none consider the effects on long term progression of disease. Quality of life and functional status are measured infrequently. While there were similarities between studies, particularly in the selection of primary outcomes that measure disease activity, other outcomes showed wide variability.

The pharmaceutical industry funded 80% of the RCTS we identified, and so it is not surprising that the frequency with which outcomes in the disease activity domain have been measured as primary or secondary outcomes reflects, to some extent, the requirements of the FDA [22], [23] and EMEA [24]–[27]. These authorities recommend, for the purpose of drug licensing and marketing authorisation, that risks and benefits of preventative therapies for children with asthma are assessed in clinical trials that measure, as primary outcomes, physiological tests of pulmonary function and clinical measures such as symptom scores. Other measures of short term disease activity, such as use of rescue medication, rate of exacerbations, and bronchial hyper-responsiveness are suggested as important outcomes. Quality of life and exercise tolerance are mentioned as additional outcomes that may provide useful information, but no clear recommendations have been made regarding the use of these outcomes [25].

It is disappointing that, despite the use of ICS in childhood asthma for more than twenty years, their effects on functional status, quality of life, and long term consequences of asthma remain largely unknown. Markers of short-term disease activity, despite their prominence in drug regulatory guidelines and popularity amongst trialists, have been shown to correlate poorly with quality of life [28]–[30], and are therefore not appropriate surrogate markers for aspects of asthma that could be of more relevance to patients.

Given that the aim of an RCT is to evaluate the safety and efficacy of interventions, and provide some assessment of whether the intervention does more good than harm, it is disappointing to note that quality of life and family outcomes are only measured in 20% of RCTs, and that the impact of disease on functional status is now measured in less than 10%. We identified no clinical trials in which the primary outcome measured these domains. In studies that have investigated which outcomes are clinically relevant to patients with other conditions, such as chronic pain [31], fibromyalgia [32], and rheumatoid arthritis [33], measures of functional status and quality of life were identified as being of great importance, and it is likely that this is the case in children with asthma.

Functional status overlaps with quality of life and disease activity. However, we feel that measures of functional status are important, distinct, markers of how asthma affects children. In clinical trials in adults with chronic illnesses, absence from work is an important outcome, and we feel that an appropriate childhood equivalent would include measures of school attendance and other activities of daily living.

It is particularly important in trials of children to assess the impact of treatments in the long term. Very few studies have attempted to study the impact of ICS on modifying or affecting the physical consequences of asthma. Two studies, the Childhood Asthma Management Program (CAMP) [20], and the Inhaled Steroid Treatment as Regular Therapy in Early Asthma (START) [21] indicated in their aims that they wished to investigate this effect. Both measured primary outcomes which we have classified as related to disease activity, although the CAMP study stated that their primary outcome (FEV1, %predicted) was a measure of ‘lung growth’. Investigators acknowledge the difficulties in assessing the impact of disease, or therapy, on ‘lung growth’ in children with asthma. It is unlikely that, in a clinical trial, a single measure will provide the best primary outcome and more methodological research is needed to identify whether longitudinal outcomes, such as rate of change in lung function measures, would be a more appropriate way of assessing lung growth. Although 24 of the studies that exclusively included children had a treatment period lasting longer than one year, only three measured outcomes after the end of the treatment period [20], [34], [35]. The other 21 studies represent missed opportunities to investigate the long term effects of ICS on progression of asthma in children, and this question should be addressed in future clinical trials.

The long term safety of treatments for asthma has recently been identified as being of particular importance to patients and clinicians [36]. Although 83% of studies that only included children assessed the safety of ICS, the quality with which long term systemic side effects of ICS were measured was variable. Of the 24 studies that lasted longer than a year, 21 measured effects on growth. None of the studies measured the final adult height attained, despite the fact that this is of most interest to children and parents. Only 14 studies measured the effect of ICS, when administered for longer than one year, on hypothalamo-pituitary-adrenal function, despite the serious and potentially fatal consequences of this type of adverse reaction [37]. We suggest that serious systemic side effects should be monitored in all clinical trials of ICS in children with asthma, so that both the benefits and risks of these drugs can be appropriately evaluated.

As well as the fact that measurement of long-term efficacy and safety outcomes is not a requirement of drug regulatory authorities, there are other reasons why they may not have been measured in the studies we identified. Diagnostic and technical problems associated with measurement of lung growth, financial cost, and problems with patient attrition also hinder the conduct of long-term studies in children with asthma. Agreement that long-term outcomes are important, amongst clinicians, patients, and researchers, could promote the conduct of such studies. There is also a need for research to identify the most appropriate long-term outcomes, and the ways in which they should be measured.

The studies we identified are comparable in terms of the population they include and the interventions which they compare, and so we feel that our finding of heterogeneity of outcome selection between studies is valid. Even though we have reviewed RCTs assessing one aspect of the treatment of childhood asthma, it is likely that our findings would be similar if we were to conduct a similar review of, for example, clinical trials of leukotriene antagonists or long acting beta_2_ agonists.

We have reviewed outcomes which have been reported rather than those which were actually measured. Outcome reporting bias in published RCT reports is common [18], [38], and in order to have evaluated exactly which outcomes had been measured it may have been more accurate to assess trial protocols. Although outcome reporting bias may lead to an underestimation of the frequency with which some outcomes were actually measured, it is unlikely that it would affect heterogeneity between studies.

Non-uniform outcome selection can make it difficult to design, interpret, and meta-analyse clinical trials [15], [16], [39], [40], and so a few collaborations have begun to address the problems of which outcomes to measure in clinical trials of a variety of paediatric and adult conditions [9], [15]. One solution is a universally-agreed core set of outcomes which should be measured, as a minimum, in all clinical trials of a specific condition. Core sets were first designed by the OMERACT group, which utilises structured consensus techniques amongst a diverse group of stakeholders and consumers. Our findings would suggest that a similar initiative in childhood asthma, with separate consideration of pre-school and older children, would make an important contribution to improving clinical research in this very prevalent, chronic disease.

### Conclusions

We have shown that outcomes in RCTs in children with asthma, mainly driven by the requirements of drug regulatory authorities, are focussed on short term disease activity, and those which may be more relevant to patients are largely overlooked. Future research must be directed towards determining the most appropriate and important outcomes to measure in these trials.

## Supporting Information

File S1 Search strategy
(0.04 MB PDF)

File S2 List of included studies
(0.32 MB PDF)
