# Supplementary material for: Outcomes in Clinical Trials of Inhaled Corticosteroids for Children with Asthma Are Narrowly Focussed on Short Term Disease Activity
Source: PLoS One. 2009 Jul 17;4(7):e6276. doi: 10.1371/journal.pone.0006276 (PMC2707602; doi:10.1371/journal.pone.0006276)
Supplement: File S1 — Search strategy (0.04 MB PDF) [file pone.0006276.s001.pdf]

Supplementary File S1 – search strategy

#1	asthma:ti,ab,kw
#2	(antiasthma OR anti-asthma):ti,ab,kw
#3	wheez*:ti,ab,kw
#4	(bronch?spas* OR bronchoconstric* OR bronchismus OR bronchiospas*):ti,ab,kw
#5	cough:ti,ab,kw
#6	(#1 OR #2 OR #3 OR #4 OR #5)
#7	(child*):ti,ab,kw
#8	(paediatric* OR pediatric):ti,ab,kw
#9	(infan*):ti,ab,kw
#10	(young*):ti,ab,kw
#11	(toddler*):ti,ab,kw
#12	bab*:ti,ab,kw
#13	(preschool or pre-school):ti,ab,kw
#14	(teenage*):ti,ab,kw
#15	(adolesce*):ti,ab,kw
#16	(#7 OR #8 OR #9 OR #10 OR #11 OR #12 OR #13 OR #14 OR #15)
#17	beclomet?asone:ti,ab,kw
#18	betamet?asone:ti,ab,kw
#19	fluticasone:ti,ab,kw
#20	budesonide:ti,ab,kw
#21	(corticosteroid* OR *corticoid*):ti,ab,kw
#22	(inhaled *steroid*):ti,ab,kw
#23	(pulmicort or azmacort or becoride or flixotide or flovent or aerobid or aerobec or qvar or vanceril or triamciclone):ti,ab,kw
#24	(#17 OR #18 OR #19 OR #20 OR #21 OR #22 OR #23)
#25	(#6 AND #16 AND #24)
