# Supplementary material for: Outcomes in Clinical Trials of Inhaled Corticosteroids for Children with Asthma Are Narrowly Focussed on Short Term Disease Activity
Source: PLoS One. 2009 Jul 17;4(7):e6276. doi: 10.1371/journal.pone.0006276 (PMC2707602; doi:10.1371/journal.pone.0006276)
Supplement: File S2 — List of included studies (0.32 MB PDF) [file pone.0006276.s002.pdf]

SUPPLEMENTARY FILE S2 – INCLUDED STUDIES

Studies exclusively including children

Author of main publication	Year	Associated publications
ACUN [1]	2005	
ADLER [2]	2001	
AGERTOFT [3]	1993	
AGERTOFT [4]	1997	
ALLEN [5]	1998	WITH MAHAJAN 1998 [6]
ALTINTAS [7]	2005	
ANDERSSON [8]	2001	
ARETS [9]	2002	
BAKER [10]	1999	WITH MELLON 2000 [11]
BARRUETO [12]	2002	
BAXTER-JONES [13]	2000	
BECKER [14]	2006	
BENNATI [15]	1989	
BERGER [16]	2006	
BERGER [17]	2005	
BISGAARD [18]	1988	
BISGAARD [19]	1990	
BISGAARD [20]	1999	WITH BISGAARD 2001 [21]
BISGAARD [22]	2004	
BONER [23]	1995	
BUKSTEIN [24]	2003	
CAMARGOS [25]	2007	
CAMP [26]	2000	WITH COVAR 2003 [27]AND BACHARIER 2004 [28]
CAMPBELL [29]	1998	
CARLSEN [30]	2005	
CHAVASSE [31]	2001	
CONNETT [32]	1993	WITH CONNETT 1993 [33]
CSERHATI [34]	2000	
DE BAETS [35]	1990	
DE BENEDICTIS [36]	2001	
DE BENEDICTIS [37]	2000	
DE BLIC [38]	1996	
DELACOURT [39]	2003	
DONIEC [40]	2004	
DOULL [41]	1995	WITH DOULL 97 [42]
EDMUNDS [43]	1994	
ESCRIBANO [44]	2006	
FARMER [45]	2000	
FERGUSON [46]	2007	

FERGUSON [47]	1999	
GARCIA GARCIA [48]	2005	
GILLMAN [49]	2002	
GUSTAFSSON [50]	1993	
HALTERMAN [51]	2004	
HODGES [52]	2005	
HOEKSTRA [53]	1996	WITH HOEKSTRA 98 [54]
HOEKX [55]	1996	
HOFHUIS [56]	2005	
HOFSTRA [57]	2000	
JAT [58]	2006	
JONASSON [59]	1998	WITH JONASSON 1999 [60], JONASSON 2000 [61], AND JONASSON 2000 [62]
KANNISTO [63]	2002	WITH KANNISTO 2000 [64] AND KANNISTO 2001 [65]
KARAMAN [66]	2007	
KARAMAN [67]	2004	
KATZ [68]	1998	
KEMP [69]	1999	SKONER 2000 [70], GEORGITIS 2001 [71]
KOH [72]	2001	
LAFORCE [73]	2000	
LEE [74]	2001	
LEFLEIN [75]	2001	WITH IRANI 02 [76]AND SKONER 00 [70]
LEFLEIN [77]	2002	WITH MURPHY 2003 [78]
LEVY [79]	2006	
LONNKVIST [80]	2001	WITH LONNKVIST 2004 [81]
LYTTLE [82]	2003	
MACKENZIE [83]	1993	
MALLOL [84]	2007	
MALONE [85]	2005	
MASPERO [86]	2001	
MELTZER [87]	1992	
MOELLER [88]	2004	
MOLLER [89]	1999	
NAYAK [90]	2002	
NIELSEN [91]	2000	
OSTROM [92]	2005	
PEDEN [93]	1998	
PEDERSEN [94]	2006	
PEDERSEN [95]	2002	WITH SZEFLER 2002 [96]
PIACENTINI [97]	1990	WITH BONER 1991 [98]
POHUNEK [99]	2006	
PRICE [100]	1995	WITH BOOTH 1996 [101]
PRICE [102]	1997	
QAQUNDAH [103]	2006	
RAO [104]	1999	WITH GREGSON 1998 [105]

ROUX [106]	2003	
RUFF [107]	2003	
RYTILA [108]	2004	
SALVATONI [109]	2000	
SEKEREL [110]	1997	
SHAPIRO [111]	2001	
SHAPIRO [112]	1998	EXTENDED BY SKONER 2000 [70]
SINGH [113]	2001	
SIMONS [114]	1997	
SORKNESS [115]	2007	
STELMACH [116]	2005	
STELMACH [117]	2002	WITH STELMACH 2004 [118]
TAL [119]	2002	
TEPER [120]	2005	
TERZANO [121]	2001	
TERZANO [122]	2000	
TINKELMAN [123]	1993	WITH TINKELMAN 1996 [124] AND BENDER 1998 [125]
VAN ESSEN [126]	1992	WITH VAN ESSEN 1994 [127], AND WAALKENS 1993 [128] AND MERKUS 1993 [129]
VANTO [130]	2004	
VERBERNE [131]	1997	
VERBERNE [132]	1998	
VERONA [133]	2003	
VISSER [134]	2001	WITH VISSER 2002 [135]
VON BERG [136]	2002	
WAALKENS [137]	1991	
WASSERMAN [138]	2006	
WENNERGREN [139]	1996	
WHITE [140]	1999	
WILLIAMS [141]	1997	
WOLTHERS [142]	1992	
YOUNG [143]	2002	
ZIELEN [144]	2006	

Studies including children and adults

Author	Year
AUBIER [145]	1999
BARANIUK [146]	1999
BENSCH [147]	2006
BERGER [148]	2002
BERNSTEIN [149]	1999
BLEECKER [150]	2000
BOULET [151]	2000

BOULET [152]	2004
BOULET [153]	2006
BOUSQUET [154]	2000
BOUSQUET [155]	2006
BUHL [156]	2006
BUSSE [157]	2001
CONDEMI [158]	1997
DI FRANCO [159]	1999
ESTRADA [160]	1991
FIREMAN [161]	2001
GALANT [162]	1996
GALANT [163]	1999
HANSEL [164]	2006
JENKINS [165]	2006
JOHANSSON [166]	2001
KARPEL [167]	2005
KIM [168]	2000
LUMRY [169]	2006
NATHAN [170]	2000
NATHAN [171]	2001
NATHAN [172]	2001
NELSON [173]	2000
NELSON [174]	2003
O CONNOR [175]	2001
PAUWELS [176]	2003
PEARLMAN [177]	2004
PEARLMAN [178]	1997
PEARLMAN [179]	1999
PINNAS [180]	2005
RABE [181]	2006
RAPHAEL [182]	2000
SCICCHITANO [183]	2004
SHAPIRO [184]	2000
SHEFFER [185]	1996
WARDLAW [186]	2004
ZUWALLACK [187]	1997
ZUWALLACK [188]	2000

Reference list

1. Acun C, Tomac N, Ermis B, Onk G (2005) Effects of inhaled corticosteroids on growth in asthmatic children: A comparison of fluticasone propionate with budesonide. Allergy and Asthma Proceedings 26: 204-206.
2. Adler LM, Anand C, Wright FGD, Barrett CF, McKeith D, et al. (2001) Efficacy and tolerability of beclomethasone dipropionate delivered by a novel multidose dry powder inhaler (Clickhaler (R)) versus a metered-dose inhaler in children with asthma. Current Therapeutic Research-Clinical and Experimental 62: 758-769.
3. Agertoft L, Pedersen S (1993) Importance of the inhalation device on the effect of budesonide. Archives of Disease in Childhood. pp. 130-133.
4. Agertoft L, Pedersen S (1997) A randomized, double-blind dose reduction study to compare the minimal effective dose of budesonide Turbuhaler and fluticasone propionate Diskhaler. Journal of Allergy and Clinical Immunology 99: 773-780.
5. Allen DB, Bronsky EA, LaForce CF, Nathan RA, Tinkelman DG, et al. (1998) Growth in asthmatic children treated with fluticasone propionate. Fluticasone Propionate Asthma Study Group. Journal of Pediatrics. pp. 472-477.
6. Mahajan P, Pearlman D, Okamoto L (1998) The effect of fluticasone propionate on functional status and sleep in children with asthma and on the quality of life of their parents. The Journal of allergy and clinical immunology. pp. 19-23.
7. Altintas DU, Karakoc GB, Can S, Yilmaz M, Kendirli SG (2005) The effects of long term use of inhaled corticosteroids on linear growth, adrenal function and bone mineral density in children. Allergol Immunopathol (Madr) 33: 204-209.
8. Andersson F, Kjellman M, Forsberg G, Moller C, Arheden L (2001) Comparison of the cost-effectiveness of budesonide and sodium cromoglycate in the management of childhood asthma in everyday clinical practice. Annals of Allergy Asthma & Immunology 86: 537-544.
9. Arets HGM, Kamps AWA, Brackel HJL, Mulder PGH, Vermue NA, et al. (2002) Children with mild asthma: do they benefit from inhaled corticosteroids? European Respiratory Journal 20: 1470-1475.
10. Baker JW, Mellon M, Wald J, Welch M, Cruz-Rivera M, et al. (1999) A multiple-dosing, placebo-controlled study of budesonide inhalation suspension given once or twice daily for treatment of persistent asthma in young children and infants. Pediatrics 103: 414-421.
11. Mellon M, Dukes E, Ollendorf D, Oster G, Cruz Rivera M (2000) Budesonide inhalation suspension improves symptom-free days and use of asthma-related services in infants and young children. Annals of Allergy, Asthma & Immunology. pp. 161.
12. Barrueto L, Mallol J, Figueroa L (2002) Beclomethasone dipropionate and salbutamol by metered dose inhaler in infants and small children with recurrent wheezing. Pediatric Pulmonology 34: 52-57.
13. Baxter-Jones AD, Helms PJ (2000) Early introduction of inhaled steroids in wheezing children presenting in primary care. A pilot study. EASE Study Group. Clinical and experimental allergy : journal of the British Society for Allergy and Clinical Immunology. pp. 1618-1626.
14. Becker AB, Kuznetsova O, Vermeulen J, Soto-Quiros ME, Young B, et al. (2006) Linear growth in prepubertal asthmatic children treated with montelukast, beclomethasone, or placebo: a 56-week randomized double-blind study. Annals of Allergy Asthma & Immunology 96: 800-807.
15. Bennati D, Piacentini GL, Peroni DG, Sette L, Testi R, et al. (1989) CHANGES IN BRONCHIAL REACTIVITY IN ASTHMATIC-CHILDREN AFTER TREATMENT WITH BECLOMETHASONE ALONE OR IN ASSOCIATION WITH SALBUTAMOL. Journal of Asthma 26: 359-364.
16. Berger WE, Milgrom H, Chervinsky P, Noonan M, Weinstein SF, et al. (2006) Effects of treatment with mometasone furoate dry powder inhaler in children with persistent asthma. Annals of Allergy Asthma & Immunology 97: 672-680.

17. Berger WE, Qaqundah PY, Blake K, Rodriguez-Santana J, Irani AM, et al. (2005) Safety of budesonide inhalation suspension in infants aged six to twelve months with mild to moderate persistent asthma or recurrent wheeze. Journal of Pediatrics 146: 91-95.
18. Bisgaard H, Damkjaer Nielsen M, Andersen B, Andersen P, Foged N, et al. (1988) Adrenal function in children with bronchial asthma treated with beclomethasone dipropionate or budesonide. The Journal of allergy and clinical immunology. pp. 1088-1095.
19. Bisgaard H, Munck SL, Nielsen JP, Petersen W, Ohlsson SV (1990) Inhaled budesonide for treatment of recurrent wheezing in early childhood. Lancet. pp. 649-651.
20. Bisgaard H, Gillies J, Groenewald M, Maden C (1999) The effect of inhaled fluticasone propionate in the treatment of young asthmatic children: a dose comparison study. American Journal of Respiratory and Critical Care Medicine. pp. 126-131.
21. Bisgaard H, Price MJ, Maden C, Olsen NA (2001) Cost-effectiveness of fluticasone propionate administered via metered-dose inhaler plus babyhaler spacer in the treatment of asthma in preschool-aged children. Chest. pp. 1835-1842.
22. Bisgaard H, Allen D, Milanowski J, Kalev I, Willits L, et al. (2004) Twelve-month safety and efficacy of inhaled fluticasone propionate in children aged 1 to 3 years with recurrent wheezing. Pediatrics 113: e87-94.
23. Boner AL, Comis A, Schiassi M, Venge P, Piacentini GL (1995) Bronchial reactivity in asthmatic children at high and low altitude. Effect of budesonide. American Journal of Respiratory and Critical Care Medicine. pp. 1194-1200.
24. Bukstein DA, Luskin AT, Bernstein A (2003) "Real-world" effectiveness of daily controller medicine in children with mild persistent asthma. Annals of Allergy Asthma & Immunology 90: 543-549.
25. Camargos P, Ibiapina C, Lasmar L, Cruz AA (2007) Obtaining concomitant control of allergic rhinitis and asthma with a nasally inhaled corticosteroid. Allergy 62: 310-316.
26. (1999) The Childhood Asthma Management Program (CAMP): design, rationale, and methods. Childhood Asthma Management Program Research Group. Controlled clinical trials. pp. 91-120.
27. Covar RA, Szefler SJ, Martin RJ, Sundstrom DA, Silkoff PE, et al. (2003) Relations between exhaled nitric oxide and measures of disease activity among children with mild-to-moderate asthma. The Journal of pediatrics. pp. 469-475.
28. Bacharier LB, Raissy HH, Wilson L, McWilliams B, Strunk RC, et al. (2004) Long-term effect of budesonide on hypothalamic-pituitary-adrenal axis function in children with mild to moderate asthma. Pediatrics. pp. 1693-1699.
29. Campbell LM, Bodalia B, Gogbashian CA, Gunn SD, Humphreys PJ, et al. (1998) Once-daily budesonide: 400 mu g once daily is as effective as 200 mu g twice daily in controlling childhood asthma. International Journal of Clinical Practice 52: 213-+.
30. Carlsen KC, Stick S, Kamin W, Cirule I, Hughes S, et al. (2005) The efficacy and safety of fluticasone propionate in very young children with persistent asthma symptoms. Respiratory Medicine. pp. 1393-1402.
31. Chavasse RJ, Bastian-Lee Y, Richter H, Hilliard T, Seddon P (2001) Persistent wheezing in infants with an atopic tendency responds to inhaled fluticasone. Archives of Disease in Childhood 85: 143-147.
32. Connett G, Lenney W (1993) Prevention of viral induced asthma attacks using inhaled budesonide. Archives of Disease in Childhood. pp. 85-87.
33. Connett GJ, Warde C, Wooler E, Lenney W (1993) Use of budesonide in severe asthmatics aged 1-3 years. Archives of Disease in Childhood. pp. 351-355.
34. Cserhati E, Spicak V (2000) The efficacy of budesonide and disodium-cromoglycate therapy in children with mild and moderate persistent bronchial asthma. International Review of Allergology & Clinical Immunology. pp. 91-98.

35. De Baets FM, Goeteyn M, Kerrebijn KF (1990) The effect of two months of treatment with inhaled budesonide on bronchial responsiveness to histamine and house-dust mite antigen in asthmatic children. The American review of respiratory disease. pp. 581-586.
36. de Benedictis FM, Teper A, Green RJ, Boner AL, Williams L, et al. (2001) Effects of 2 inhaled corticosteroids on growth: results of a randomized controlled trial. Archives of pediatrics & adolescent medicine. pp. 1248-1254.
37. De Benedictis FM, Boner A, Cavagni G, Caffarelli C, Ferraro L, et al. (2000) Treating asthma in children with beclomethasone dipropionate: Pulvinal versus Diskhaler. Journal of aerosol medicine : the official journal of the International Society for Aerosols in Medicine. pp. 35-41.
38. de Blic J, Delacourt C, Le Bourgeois M, Mahut B, Ostinelli J, et al. (1996) Efficacy of nebulized budesonide in treatment of severe infantile asthma: a double-blind study. The Journal of allergy and clinical immunology. pp. 14-20.
39. Delacourt C, Dutau G, Lefrançois G, Clerson P, Beclospin Clinical Development G (2003) Comparison of the efficacy and safety of nebulized beclometasone dipropionate and budesonide in severe persistent childhood asthma. Respiratory Medicine. pp. S27-33.
40. Doniec Z, Pierzchala-Koziec K, Tomalak W, Nowak D, Kurzawa R (2004) Powder budesonide decreases plasma level of native and cryptic met-enkephalin in asthmatic children. International Review of Allergology & Clinical Immunology. pp. 105-109.
41. Doull IJ, Freezer NJ, Holgate ST (1995) Growth of prepubertal children with mild asthma treated with inhaled beclomethasone dipropionate. American Journal of Respiratory and Critical Care Medicine. pp. 1715-1719.
42. Doull IJ, Lampe FC, Smith S, Schreiber J, Freezer NJ, et al. (1997) Effect of inhaled corticosteroids on episodes of wheezing associated with viral infection in school age children: randomised double blind placebo controlled trial. BMJ (Clinical research ed). pp. 858-862.
43. Edmunds AT, Goldberg RS, Duper B, Devichand P, Follows RMA (1994) A comparison of budesonide 800 micro-grams and 400 micro-grams daily via turbohaler with disodium cromoglycate via Spinhaler for asthma prophylaxis in children. British Journal of Clinical Research. pp. 11-23.
44. Escribano A, Tutuncu A, Löhr I, Carlholm M, Polanowski T (2006) Clinical comparability between the CFC and HFA budesonide pressurised metered-dose inhalers in paediatric patients with asthma: a randomised controlled trial. Current medical research and opinion. pp. 1085-1092.
45. Farmer IS, Middle M, Savic J, Perri VL, Herdman MJ (2000) Therapeutic equivalence of inhaled beclomethasone dipropionate with CFC and non-CFC (HFA 134a) propellants both delivered via the Easibreathe inhaler for the treatment of paediatric asthma. Respiratory Medicine. pp. 57-63.
46. Ferguson AC, Van Bever HP, Teper AM, Lasytsya O, Goldfrad CH, et al. (2007) A comparison of the relative growth velocities with budesonide and fluticasone propionate in children with asthma. Respiratory Medicine. pp. 118-129.
47. Ferguson AC, Spier S, Manjra A, Versteegh FG, Mark S, et al. (1999) Efficacy and safety of high-dose inhaled steroids in children with asthma: a comparison of fluticasone propionate with budesonide. The Journal of pediatrics. pp. 422-427.
48. Garcia Garcia ML, Wahn U, Gilles L, Swern A, Tozzi CA, et al. (2005) Montelukast, compared with fluticasone, for control of asthma among 6- to 14-year-old patients with mild asthma: the MOSAIC study. Pediatrics. pp. 360-369.
49. Gillman SA, Anolik R, Schenkel E, Newman K (2002) One-year trial on safety and normal linear growth with flunisolide HFA in children with asthma. Clinical pediatrics. pp. 333-340.
50. Gustafsson P, Tsanakas J, Gold M, Primhak R, Radford M, et al. (1993) Comparison of the efficacy and safety of inhaled fluticasone propionate 200 microg/day with inhaled beclomethasone dipropionate 400 microg/day in mild and moderate asthma. Arch Dis Child. pp. 206-211.

51. Halterman JS, Szilagyi PG, Yoos HL, Conn KM, Kaczorowski JM, et al. (2004) Benefits of a school-based asthma treatment program in the absence of secondhand smoke exposure: results of a randomized clinical trial. Archives of pediatrics & adolescent medicine. pp. 460-467.
52. Hodges IGC, Netherway TA (2005) Once-daily fluticasone propionate is as effective as twice-daily treatment in stable, mild-to-moderate childhood asthma. Clinical Drug Investigation. pp. 13-22.
53. Hoekstra MO, Grol MH, Bouman K, Stijnen T, Köeter GH, et al. (1996) Fluticasone propionate in children with moderate asthma. American Journal of Respiratory and Critical Care Medicine. pp. 1039-1044.
54. Hoekstra MO, Grol MH, Hovenga H, Bouman K, Stijnen T, et al. (1998) Eosinophil and mast cell parameters in children with stable moderate asthma. Pediatric allergy and immunology : official publication of the European Society of Pediatric Allergy and Immunology. pp. 143-149.
55. Hoekx JC, Hedlin G, Pedersen W, Sorva R, Hollingworth K, et al. (1996) Fluticasone propionate compared with budesonide: a double-blind trial in asthmatic children using powder devices at a dosage of 400 microg x day(-1). The European respiratory journal : official journal of the European Society for Clinical Respiratory Physiology. pp. 2263-2272.
56. Hofhuis W, van der Wiel EC, Nieuwhof EM, Hop WC, Affourtit MJ, et al. (2005) Efficacy of fluticasone propionate on lung function and symptoms in wheezy infants. American Journal of Respiratory and Critical Care Medicine. pp. 328-333.
57. Hofstra WB, Neijens HJ, Duiverman EJ, Kouwenberg JM, Mulder PG, et al. (2000) Dose-responses over time to inhaled fluticasone propionate treatment of exercise- and methacholine-induced bronchoconstriction in children with asthma. Pediatric Pulmonology. pp. 415-423.
58. Jat GC, Mathew JL, Singh M (2006) Treatment with 400 microg of inhaled budesonide vs 200 microg of inhaled budesonide and oral montelukast in children with moderate persistent asthma: randomized controlled trial. Annals of allergy, asthma & immunology : official publication of the American College of Allergy, Asthma, & Immunology. pp. 397-401.
59. Jónasson G, Carlsen KH, Blomqvist P (1998) Clinical efficacy of low-dose inhaled budesonide once or twice daily in children with mild asthma not previously treated with steroids. The European respiratory journal : official journal of the European Society for Clinical Respiratory Physiology. pp. 1099-1104.
60. Jónasson G, Carlsen KH, Sødal A, Jonasson C, Mowinckel P (1999) Patient compliance in a clinical trial with inhaled budesonide in children with mild asthma. The European respiratory journal : official journal of the European Society for Clinical Respiratory Physiology. pp. 150-154.
61. Jónasson G, Carlsen KH, Hultquist C (2000) Low-dose budesonide improves exercise-induced bronchospasm in schoolchildren. Pediatric allergy and immunology : official publication of the European Society of Pediatric Allergy and Immunology. pp. 120-125.
62. Jónasson G, Carlsen KH, Mowinckel P (2000) Asthma drug adherence in a long term clinical trial. Archives of Disease in Childhood. pp. 330-333.
63. Kannisto S, Voutilainen R, Remes K, Korppi M (2002) Efficacy and safety of inhaled steroid and cromone treatment in school-age children: a randomized pragmatic pilot study. Pediatric allergy and immunology : official publication of the European Society of Pediatric Allergy and Immunology. pp. 24-30.
64. Kannisto S, Korppi M, Remes K, Voutilainen R (2000) Adrenal suppression, evaluated by a low dose adrenocorticotropin test, and growth in asthmatic children treated with inhaled steroids. The Journal of clinical endocrinology and metabolism. pp. 652-657.
65. Kannisto S, Korppi M, Remes K, Voutilainen R (2001) Serum dehydroepiandrosterone sulfate concentration as an indicator of adrenocortical suppression in asthmatic children treated with inhaled steroids. The Journal of clinical endocrinology and metabolism. pp. 4908-4912.
66. Karaman O, Arli O, Uzuner N, Islekel H, Babayigit A, et al. (2007) The effectiveness of asthma therapy alternatives and evaluating the effectivity of asthma therapy by interleukin-13 and

interferon gamma levels in children. Allergy and asthma proceedings : the official journal of regional and state allergy societies. pp. 204-209.
67. Karaman O, Sünneli L, Uzuner N, Islekel H, Turgut CS, et al. (2004) Evaluation of montelukast in 8 to 14 year old children with mild persistent asthma and compared with inhaled corticosteroids. Allergologia et immunopathologia. pp. 21-27.
68. Katz Y, Lebas FX, Medley HV, Robson R (1998) Fluticasone propionate 50 micrograms BID versus 100 micrograms BID in the treatment of children with persistent asthma Fluticasone Propionate Study Group. Clinical Therapeutics. pp. 424-437.
69. Kemp JP, Skoner DP, Szefler SJ, Walton-Bowen K, Cruz-Rivera M, et al. (1999) Once-daily budesonide inhalation suspension for the treatment of persistent asthma in infants and young children. Annals of allergy, asthma & immunology : official publication of the American College of Allergy, Asthma, & Immunology. pp. 231-239.
70. Skoner DP, Szefler SJ, Welch M, Walton-Bowen K, Cruz-Rivera M, et al. (2000) Longitudinal growth in infants and young children treated with budesonide inhalation suspension for persistent asthma. The Journal of allergy and clinical immunology. pp. 259-268.
71. Georgitis JW, McWilliams B, Cruz-rivera M, Fitzpatrick S, Smith JA (2001) Effective once-daily administration of Budesonide inhalation suspension by nebulizer with facemasks or mouthpieces for persistent asthma in infants and young children. Pediatric Asthma, Allergy & Immunology. pp. 3-13.
72. Koh YY, Sun YH, Lim HS, Kim CK, Hong SJ (2001) Effect of inhaled budesonide on bronchial hyperresponsiveness in adolescents with clinical remission of asthma. Chest. pp. 1140-1146.
73. LaForce CF, Pearlman DS, Ruff ME, Silvers WS, Weinstein SW, et al. (2000) Efficacy and safety of dry powder fluticasone propionate in children with persistent asthma. Annals of allergy, asthma & immunology : official publication of the American College of Allergy, Asthma, & Immunology. pp. 407-415.
74. Lee TL, Adler L, McLaren G, Rossetti A, Cantini L (2001) Assessment of efficacy and systemic safety of a new chlorofluorocarbon-free formulation of inhaled beclomethasone dipropionate in asthmatic children. Pediatric Asthma Allergy & Immunology. pp. 133-143.
75. Leflein JG, Gawchik SM, Galant SP, Lyzell E, Young M, et al. (2001) Safety of budesonide inhalation suspension (Pulmicort Respules) after up to 52 weeks of treatment in infants and young children with persistent asthma. Allergy and asthma proceedings : the official journal of regional and state allergy societies. pp. 359-366.
76. Irani AM, Cruz-Rivera M, Fitzpatrick S, Hoag J, Smith JA (2002) Effects of budesonide inhalation suspension on hypothalamic-pituitary-adrenal-axis function in infants and young children with persistent asthma. Annals of allergy, asthma & immunology : official publication of the American College of Allergy, Asthma, & Immunology. pp. 306-312.
77. Leflein JG, Szefler SJ, Murphy KR, Fitzpatrick S, Cruz Rivera M, et al. (2002) Nebulized Budesonide inhalation suspension compared with Cromlyn Sodium Nebulizer solution for asthma in young children: results of a randomised outcomes trial. Pediatrics. pp. 866-872.
78. Murphy KR, Fitzpatrick S, Cruz-Rivera M, Miller CJ, Parasuraman B (2003) Effects of budesonide inhalation suspension compared with cromolyn sodium nebulizer solution on health status and caregiver quality of life in childhood asthma. Pediatrics. pp. e212-219.
79. Levy AL, Given JT, Weinstein SF, Kent, Jr., Ef, et al. (2006) Efficacy and safety of fluticasone propionate hydrofluoroalkane inhalation aerosol in four- to eleven-year-old children with asthma: A randomized, double-blinded, placebo-controlled study. Pediatric Asthma, Allergy and Immunology. pp. 106-117.
80. Lönnkvist K, Hellman C, Lundahl J, Halldén G, Hedlin G (2001) Eosinophil markers in blood, serum, and urine for monitoring the clinical course in childhood asthma: impact of budesonide treatment and withdrawal. The Journal of allergy and clinical immunology. pp. 812-817.

81. Lönnkvist K, Anderson M, Hedlin G, Svartengren M (2004) Exhaled NO and eosinophil markers in blood, nasal lavage and sputum in children with asthma after withdrawal of budesonide. Pediatric allergy and immunology : official publication of the European Society of Pediatric Allergy and Immunology. pp. 351-358.
82. Lyttle B, Gilles J, Panov M, Emeryk A, Wixon C (2003) Fluticasone propionate 100 microg bid using a non-CFC propellant, HFA 134a, in asthmatic children. Canadian respiratory journal : journal of the Canadian Thoracic Society. pp. 103-109.
83. MacKenzie CA, Weinberg EG, Tabachnik E, Taylor M, Havnen J, et al. (1993) A placebo controlled trial of fluticasone propionate in asthmatic children. European journal of pediatrics. pp. 856-860.
84. Mallol J, Aguirre V (2007) Once versus twice daily budesonide metered-dose inhaler in children with mild to moderate asthma: effect on symptoms and bronchial responsiveness. Allergologia et immunopathologia. pp. 25-31.
85. Malone R, LaForce C, Nimmagadda S, Schoaf L, House K, et al. (2005) The safety of twice-daily treatment with fluticasone propionate and salmeterol in pediatric patients with persistent asthma. Annals of allergy, asthma & immunology : official publication of the American College of Allergy, Asthma, & Immunology. pp. 66-71.
86. Maspero JF, Dueñas-Meza E, Volovitz B, Pinacho Daza C, Kosa L, et al. (2001) Oral montelukast versus inhaled beclomethasone in 6- to 11-year-old children with asthma: results of an open-label extension study evaluating long-term safety, satisfaction, and adherence with therapy. Current medical research and opinion. pp. 96-104.
87. Meltzer EO, Orgel HA, Ellis EF, Eigen HN, Hemstreet MP (1992) Long-term comparison of three combinations of albuterol, theophylline, and beclomethasone in children with chronic asthma. The Journal of allergy and clinical immunology. pp. 2-11.
88. Moeller A, Franklin P, Hall GL, Turner S, Straub D, et al. (2004) Inhaled fluticasone dipropionate decreases levels of nitric oxide in recurrenty wheezy infants. Pediatric Pulmonology. pp. 250-255.
89. Möller C, Strömberg L, Oldaeus G, Arweström E, Kjellman M (1999) Efficacy of once-daily versus twice-daily administration of budesonide by Turbuhaler(R) in children with stable asthma. Pediatric Pulmonology. pp. 337-343.
90. Nayak A, Lanier R, Weinstein S, Stampone P, Welch M (2002) Efficacy and safety of beclomethasone dipropionate extrafine aerosol in childhood asthma: a 12-week, randomized, double-blind, placebo-controlled study. Chest. pp. 1956-1965.
91. Nielsen KG, Bisgaard H (2000) The effect of inhaled budesonide on symptoms, lung function, and cold air and methacholine responsiveness in 2- to 5-year-old asthmatic children. American Journal of Respiratory and Critical Care Medicine. pp. 1500-1506.
92. Ostrom NK, Decotiis BA, Lincourt WR, Edwards LD, Hanson KM, et al. (2005) Comparative efficacy and safety of low-dose fluticasone propionate and montelukast in children with persistent asthma. The Journal of pediatrics. pp. 213-220.
93. Peden DB, Berger WE, Noonan MJ, Thomas MR, Hendricks VL, et al. (1998) Inhaled fluticasone propionate delivered by means of two different multidose powder inhalers is effective and safe in a large pediatric population with persistent asthma. The Journal of allergy and clinical immunology. pp. 32-38.
94. Pedersen S, Garcia Garcia ML, Manjra A, Theron I, Engelstätter R (2006) A comparative study of inhaled ciclesonide 160 microg/day and fluticasone propionate 176 microg/day in children with asthma. Pediatric Pulmonology. pp. 954-961.
95. Pedersen S, Warner J, Wahn U, Staab D, Le Bourgeois M, et al. (2002) Growth, systemic safety, and efficacy during 1 year of asthma treatment with different beclomethasone dipropionate formulations: an open-label, randomized comparison of extrafine and conventional aerosols in children. Pediatrics. pp. e92.

96. Szefler SJ, Warner J, Staab D, Wahn U, Le Bourgeois M, et al. (2002) Switching from conventional to extrafine aerosol beclomethasone dipropionate therapy in children: a 6-month, open-label, randomized trial. The Journal of allergy and clinical immunology. pp. 45-50.
97. Piacentini GL, Sette L, Peroni DG, Bonizzato C, Bonetti S, et al. (1990) Double-blind evaluation of effectiveness and safety of flunisolide aerosol for treatment of bronchial asthma in children. Allergy. pp. 612-616.
98. Boner AL, Piacentini GL, Bonizzato C, Dattoli V, Sette L (1991) Effect of inhaled beclomethasone dipropionate on bronchial hyperreactivity in asthmatic children during maximal allergen exposure. Pediatric Pulmonology. pp. 2-5.
99. Pohunek P, Kuna P, Jorup C, De Boeck K (2006) Budesonide/formoterol improves lung function compared with budesonide alone in children with asthma. Pediatric allergy and immunology : official publication of the European Society of Pediatric Allergy and Immunology. pp. 458-465.
100. Price JF, Weller PH (1995) Comparison of fluticasone propionate and sodium cromoglycate for the treatment of childhood asthma (an open parallel group study). Respiratory Medicine. pp. 363-368.
101. Booth PC, Wells NE, Morrison AK (1996) A comparison of the cost effectiveness of alternative prophylactic therapies in childhood asthma. PharmacoEconomics. pp. 262-268.
102. Price JF, Russell G, Hindmarsh PC, Weller P, Heaf DP, et al. (1997) Growth during one year of treatment with fluticasone propionate or sodium cromoglycate in children with asthma. Pediatric Pulmonology. pp. 178-186.
103. Qaqundah PY, Sugerman RW, Ceruti E, Maspero JF, Kleha JF, et al. (2006) Efficacy and safety of fluticasone propionate hydrofluoroalkane inhalation aerosol in pre-school-age children with asthma: a randomized, double-blind, placebo-controlled study. The Journal of pediatrics. pp. 663-670.
104. Rao R, Gregson RK, Jones AC, Taylor PA, Murrills AJ, et al. (1997) Effects of inhaled corticosteroids on growth and bone turnover in childhood asthma: comparison of fluticasone propionate and beclomethasone dipropionate [abstract]. European Respiratory Journal - Supplement. pp. 24s.
105. Gregson RK, Rao R, Murrills AJ, Taylor PA, Warner JO (1998) Effect of inhaled corticosteroids on bone mineral density in childhood asthma: comparison of fluticasone propionate with beclomethasone dipropionate. Osteoporosis international : a journal established as result of cooperation between the European Foundation for Osteoporosis and the National Osteoporosis Foundation of the USA. pp. 418-422.
106. Roux C, Kolta S, Desfougères JL, Minini P, Bidat E (2003) Long-term safety of fluticasone propionate and nedocromil sodium on bone in children with asthma. Pediatrics. pp. e706-713.
107. Ruff ME, Szefler SJ, Meltzer EO, Berger WE (2003) Efficacy and safety of extrafine beclomethasone dipropionate aerosol therapy in children with asthma: a twelve-week placebo-controlled trial. Pediatric Asthma Allergy & Immunology. pp. 1-13.
108. Rytilä P, Pelkonen AS, Metso T, Nikander K, Haahtela T, et al. (2004) Induced sputum in children with newly diagnosed mild asthma: the effect of 6 months of treatment with budesonide or disodium cromoglycate. Allergy. pp. 839-844.
109. Salvatoni A, Nosetti L, Broggini M, Nespoli L (2000) Body composition and growth in asthmatic children treated with inhaled steroids. Annals of allergy, asthma & immunology : official publication of the American College of Allergy, Asthma, & Immunology. pp. 221-226.
110. Sekerel BE, Tuncer A, Saraçlar Y, Adalio, lu G (1997) Inhaled budesonide reduces lung hyperinflation in children with asthma. Acta paediatrica (Oslo, Norway : 1992). pp. 932-936.
111. Shapiro GG, Mendelson LM, Pearlman DS (2001) Once-daily budesonide inhalation powder (Pulmicort Turbuhaler) maintains pulmonary function and symptoms of asthmatic children

previously receiving inhaled corticosteroids. Annals of allergy, asthma & immunology : official publication of the American College of Allergy, Asthma, & Immunology. pp. 633-640.
112. Shapiro G, Mendelson L, Kraemer MJ, Cruz-Rivera M, Walton-Bowen K, et al. (1998) Efficacy and safety of budesonide inhalation suspension (Pulmicort Respules) in young children with inhaled steroid-dependent, persistent asthma. The Journal of allergy and clinical immunology. pp. 789-796.
113. Singh M, Kumar L (2001) Randomized comparison of a dry powder inhaler and metered dose inhaler with spacer in management of children with asthma. Indian pediatrics. pp. 24-28.
114. Simons FE (1997) A comparison of beclomethasone, salmeterol, and placebo in children with asthma. Canadian Beclomethasone Dipropionate-Salmeterol Xinafoate Study Group. The New England journal of medicine. pp. 1659-1665.
115. Sorkness CA, Lemanske RF, Mauger DT, Boehmer SJ, Chinchilli VM, et al. (2007) Long-term comparison of 3 controller regimens for mild-moderate persistent childhood asthma: the Pediatric Asthma Controller Trial. The Journal of allergy and clinical immunology. pp. 64-72.
116. Stelmach I, Grzelewski T, Jerzynska J, Kuna P (2005) A randomized, double-blind trial on the effect of treatment with montelukast, budesonide, montelukast with budesonide, formoterol with budesonide on lung function and clinical symptoms in children with asthma [abstract]. Journal of Allergy and Clinical Immunology. pp. S151.
117. Stelmach I, Jerzynska J, Kuna P (2002) A randomized, double-blind trial of the effect of glucocorticoid, antileukotriene and beta-agonist treatment on IL-10 serum levels in children with asthma. Clinical and experimental allergy : journal of the British Society for Allergy and Clinical Immunology. pp. 264-269.
118. Stelmach I, Majak P, Jerzynska J, Stelmach W, Kuna P (2004) Comparative effect of triamcinolone, nedocromil and montelukast on asthma control in children: A randomized pragmatic study. Pediatric allergy and immunology : official publication of the European Society of Pediatric Allergy and Immunology. pp. 359-364.
119. Tal A, Simon G, Vermeulen JH, Petru V, Cobos N, et al. (2002) Budesonide/formoterol in a single inhaler versus inhaled corticosteroids alone in the treatment of asthma. Pediatric Pulmonology. pp. 342-350.
120. Teper AM, Kofman CD, Szulman GA, Vidaurreta SM, Maffey AF (2005) Fluticasone improves pulmonary function in children under 2 years old with risk factors for asthma. American Journal of Respiratory and Critical Care Medicine. pp. 587-590.
121. Terzano C, Barkai L, Cremonesi G (2001) Corticosteroids administered by nebulization to children with bronchial asthma. Advances in therapy. pp. 253-260.
122. Terzano C, Allegra L, Barkai L, Cremonesi G (2001) Beclomethasone dipropionate versus budesonide inhalation suspension in children with mild to moderate persistent asthma. European review for medical and pharmacological sciences. pp. 17-24.
123. Tinkelman DG, Reed CE, Nelson HS, Offord KP (1993) Aerosol beclomethasone dipropionate compared with theophylline as primary treatment of chronic, mild to moderately severe asthma in children. Pediatrics. pp. 64-77.
124. Tinkelman D (1996) Theophylline therapy for children with asthma. European Respiratory Review. pp. 79-83.
125. Bender BG, Iklé DN, DuHamel T, Tinkelman D (1998) Neuropsychological and behavioral changes in asthmatic children treated with beclomethasone dipropionate versus theophylline. Pediatrics. pp. 355-360.
126. van Essen-Zandvliet EE, Hughes MD, Waalkens HJ, Duiverman EJ, Pocock SJ, et al. (1992) Effects of 22 months of treatment with inhaled corticosteroids and/or beta-2-agonists on lung function, airway responsiveness, and symptoms in children with asthma. The Dutch Chronic Non-specific Lung Disease Study Group. The American review of respiratory disease. pp. 547-554.

127. van Essen-Zandvliet EE, Hughes MD, Waalkens HJ, Duiverman EJ, Kerrebijn KF (1994) Remission of childhood asthma after long-term treatment with an inhaled corticosteroid (budesonide): can it be achieved? Dutch CNSLD Study Group. The European respiratory journal : official journal of the European Society for Clinical Respiratory Physiology. pp. 63-68.
128. Waalkens HJ, van Essen-Zandvliet EE, Gerritsen J, Duiverman EJ, Kerrebijn KF, et al. (1993) The effect of an inhaled corticosteroid (budesonide) on exercise-induced asthma in children. Dutch CNSLD Study Group. The European respiratory journal : official journal of the European Society for Clinical Respiratory Physiology. pp. 652-656.
129. Merkus PJ, van Essen-Zandvliet EE, Duiverman EJ, van Houwelingen HC, Kerrebijn KF, et al. (1993) Long-term effect of inhaled corticosteroids on growth rate in adolescents with asthma. Pediatrics. pp. 1121-1126.
130. Vanto T, Hämäläinen KM, Vahteristo M, Wille S, Njå F, et al. (2004) Comparison of two budesonide dry powder inhalers in the treatment of asthma in children. Journal of aerosol medicine : the official journal of the International Society for Aerosols in Medicine. pp. 15-24.
131. Verberne AA, Frost C, Roorda RJ, van der Laag H, Kerrebijn KF (1997) One year treatment with salmeterol compared with beclomethasone in children with asthma. The Dutch Paediatric Asthma Study Group. American Journal of Respiratory and Critical Care Medicine. pp. 688-695.
132. Verberne AA, Frost C, Duiverman EJ, Grol MH, Kerrebijn KF (1998) Addition of salmeterol versus doubling the dose of beclomethasone in children with asthma. The Dutch Asthma Study Group. American Journal of Respiratory and Critical Care Medicine. pp. 213-219.
133. Verona E, Petrov D, Cserhati E, Hofman J, Geppe N, et al. (2003) Fluticasone propionate in asthma: a long term dose comparison study. Archives of Disease in Childhood. pp. 503-509.
134. Visser MJ, Postma DS, Arends LR, de Vries TW, Duiverman EJ, et al. (2001) One-year treatment with different dosing schedules of fluticasone propionate in childhood asthma. Effects on hyperresponsiveness, lung function, and height. American Journal of Respiratory and Critical Care Medicine. pp. 2073-2077.
135. Visser MJ, Postma DS, Brand PL, Arends LR, Duiverman EJ, et al. (2002) Influence of different dosage schedules of inhaled fluticasone propionate on peripheral blood cytokine concentrations in childhood asthma. Clinical and experimental allergy : journal of the British Society for Allergy and Clinical Immunology. pp. 1497-1503.
136. von Berg A, Gappa M, Steinkamp G, Flit DSG (2002) Fluticasone for the treatment of symptomatic bronchial asthma in children treated with sodium cromogylate--a prospective, randomised trial. European journal of medical research. pp. 257-264.
137. Waalkens HJ, Gerritsen J, Koëter GH, Krouwels FH, van Aalderen WM, et al. (1991) Budesonide and terbutaline or terbutaline alone in children with mild asthma: effects on bronchial hyperresponsiveness and diurnal variation in peak flow. Thorax. pp. 499-503.
138. Wasserman RL, Baker JW, Kim KT, Blake KV, Scott CA, et al. (2006) Efficacy and safety of inhaled fluticasone propionate chlorofluorocarbon in 2- to 4-year-old patients with asthma: results of a double-blind, placebo-controlled study. Annals of allergy, asthma & immunology : official publication of the American College of Allergy, Asthma, & Immunology. pp. 808-818.
139. Wennergren G, Nordvall SL, Hedlin G, Möller C, Wille S, et al. (1996) Nebulized budesonide for the treatment of moderate to severe asthma in infants and toddlers. Acta paediatrica (Oslo, Norway : 1992). pp. 183-189.
140. White MV, Cruz-Rivera M, Walton-Bowen K (1999) The efficacy and safety of budesonide inhalation suspension: a nebulizable corticosteroid for persistent asthma in infants and young children. Family medicine. pp. 337-345.
141. Williams J, Richards KA (1997) Ease of handling and clinical efficacy of fluticasone propionate Accuhaler/Diskus inhaler compared with the Turbohaler inhaler in paediatric patients. UK Study Group. The British journal of clinical practice. pp. 147-153.

142. Wolthers OD, Pedersen S (1992) Controlled study of linear growth in asthmatic children during treatment with inhaled glucocorticosteroids. Pediatrics. pp. 839-842.
143. Young YK, Park Y, Chang KK (2002) Maximal airway response in adolescents with long-term asthma remission and persisting airway hypersensitivity: Its profile and the effect of inhaled corticosteroids. Chest. pp. 1214-1221.
144. Zielen S, Rose MA, Bez C, Jarisch A, Reichenbach J, et al. (2006) Effectiveness of budesonide nebulising suspension compared to disodium cromoglycate in early childhood asthma. Current medical research and opinion. pp. 367-373.
145. Aubier M, Pieters WR, Schlösser NJ, Steinmetz KO (1999) Salmeterol/fluticasone propionate (50/500 microg) in combination in a Diskus inhaler (Seretide) is effective and safe in the treatment of steroid-dependent asthma. Respiratory Medicine. pp. 876-884.
146. Baraniuk J, Murray JJ, Nathan RA, Berger WE, Johnson M, et al. (1999) Fluticasone alone or in combination with salmeterol vs triamcinolone in asthma. Chest. pp. 625-632.
147. Bensch GW, Prenner B, Berkowitz R, Galant S, Ramsdell J, et al. (2006) Once-daily evening administration of mometasone furoate in asthma treatment initiation. Annals of allergy, asthma & immunology : official publication of the American College of Allergy, Asthma, & Immunology. pp. 533-540.
148. Berger WE, Ford LB, Mahr T, Nathan RA, Crim C, et al. (2002) Efficacy and safety of fluticasone propionate 250 microg administered once daily in patients with persistent asthma treated with or without inhaled corticosteroids. Annals of allergy, asthma & immunology : official publication of the American College of Allergy, Asthma, & Immunology. pp. 393-399.
149. Bernstein DI, Berkowitz RB, Chervinsky P, Dvorin DJ, Finn AF, et al. (1999) Dose-ranging study of a new steroid for asthma: mometasone furoate dry powder inhaler. Respiratory Medicine. pp. 603-612.
150. Bleecker ER, Welch MJ, Weinstein SF, Kalberg C, Johnson M, et al. (2000) Low-dose inhaled fluticasone propionate versus oral zafirlukast in the treatment of persistent asthma. The Journal of allergy and clinical immunology. pp. 1123-1129.
151. Boulet LP, Cowie RL, Negro RD, Brett W, Gold M, et al. (2000) Comparison of once- with twice-daily dosing of fluticasone propionate in mild and moderate asthma. Canadian respiratory journal : journal of the Canadian Thoracic Society. pp. 239-247.
152. Boulet LP, Cartier A, Ernst P, Larivée P, Laviolette M (2004) Safety and efficacy of HFA-134a beclomethasone dipropionate extra-fine aerosol over six months. Canadian respiratory journal : journal of the Canadian Thoracic Society. pp. 123-130.
153. Boulet LP, Drollmann A, Magyar P, Timar M, Knight A, et al. (2006) Comparative efficacy of once-daily ciclesonide and budesonide in the treatment of persistent asthma. Respiratory Medicine. pp. 785-794.
154. Bousquet J, D'Urzo A, Hebert J, Barraza CH, Boulet LP, et al. (2000) Comparison of the efficacy and safety of mometasone furoate dry powder inhaler to budesonide Turbuhaler. The European respiratory journal : official journal of the European Society for Clinical Respiratory Physiology. pp. 808-816.
155. Bousquet J, Aubier M, Sastre J, Izquierdo JL, Adler LM, et al. (2006) Comparison of roflumilast, an oral anti-inflammatory, with beclomethasone dipropionate in the treatment of persistent asthma. Allergy. pp. 72-78.
156. Buhl R, Vinkler I, Magyar P, Györi Z, Rybacki C, et al. (2006) Comparable efficacy of ciclesonide once daily versus fluticasone propionate twice daily in asthma. Pulmonary pharmacology & therapeutics. pp. 404-412.
157. Busse W, Raphael GD, Galant S, Kalberg C, Goode-Sellers S, et al. (2001) Low-dose fluticasone propionate compared with montelukast for first-line treatment of persistent asthma: a randomized clinical trial. The Journal of allergy and clinical immunology. pp. 461-468.
158. Condemi JJ, Chervinsky P, Goldstein MF, Ford LB, Berger WE, et al. (1997) Fluticasone propionate powder administered through Diskhaler versus triamcinolone acetonide aerosol

administered through metered-dose inhaler in patients with persistent asthma. The Journal of allergy and clinical immunology. pp. 467-474.

159. Di Franco A, Giannini D, Bacci E, Dente FL, Vagaggini B, et al. (1999) Comparison of different long-term asthma treatments in subjects with mild-to-moderate asthma. Monaldi archives for chest disease = Archivio Monaldi per le malattie del torace / Fondazione clinica del lavoro, IRCCS [and] Istituto di clinica tisiologica e malattie apparato respiratorio, Università di Napoli, Secondo ateneo. pp. 390-393.
160. Estrada Rodríguez JL, Belchi Hernández J, Florido López JF, López Serrano C, Martínez Alzamora F, et al. (1991) Short-term treatment of asthma with budesonide versus placebo. Journal of investigational allergology & clinical immunology : official organ of the International Association of Asthmology (INTERASMA) and Sociedad Latinoamericana de Alergia e Inmunología. pp. 266-270.
161. Fireman P, Prenner BM, Vincken W, Demedts M, Mol SJ, et al. (2001) Long-term safety and efficacy of a chlorofluorocarbon-free beclomethasone dipropionate extrafine aerosol. Annals of allergy, asthma & immunology : official publication of the American College of Allergy, Asthma, & Immunology. pp. 557-565.
162. Galant SP, Lawrence M, Meltzer EO, Tomasko M, Baker KA, et al. (1996) Fluticasone propionate compared with theophylline for mild-to-moderate asthma. Annals of allergy, asthma & immunology : official publication of the American College of Allergy, Asthma, & Immunology. pp. 112-118.
163. Galant SP, van Bavel J, Finn A, Gross G, Pleskow W, et al. (1999) Diskus and diskhaler: efficacy and safety of fluticasone propionate via two dry powder inhalers in subjects with mild-to-moderate persistent asthma. Annals of allergy, asthma & immunology : official publication of the American College of Allergy, Asthma, & Immunology. pp. 273-280.
164. Hansel TT, Benezet O, Kafé H, Ponitz HH, Cheung D, et al. (2006) A multinational, 12-week, randomized study comparing the efficacy and tolerability of ciclesonide and budesonide in patients with asthma. Clinical therapeutics. pp. 906-920.
165. Jenkins C, Kolarikova R, Kuna P, Caillaud D, Sanchis J, et al. (2006) Efficacy and safety of high-dose budesonide/formoterol (Symbicort) compared with budesonide administered either concomitantly with formoterol or alone in patients with persistent symptomatic asthma. Respirology (Carlton, Vic). pp. 276-286.
166. Johansson G, McIvor RA, D'Ambrosio FP, Gratziou C, James MH (2001) Comparison of salmeterol/fluticasone propionate combination with budesonide in patients with mild-to-moderate asthma. Clinical Drug Investigation. pp. 633-642.
167. Karpel JP, Busse WW, Noonan MJ, Monahan ME, Lutsky B, et al. (2005) Effects of mometasone furoate given once daily in the evening on lung function and symptom control in persistent asthma. The Annals of pharmacotherapy. pp. 1977-1983.
168. Kim KT, Ginchansky EJ, Friedman BF, Srebro S, Pepsin PJ, et al. (2000) Fluticasone propionate versus zafirlukast: effect in patients previously receiving inhaled corticosteroid therapy. Annals of allergy, asthma & immunology : official publication of the American College of Allergy, Asthma, & Immunology. pp. 398-406.
169. Lumry WR, Conway MM, LaForce CF, Pearlman DS, Scott CA, et al. (2006) Fluticasone propionate hydrofluoroalkane inhalation aerosol in patients receiving inhaled corticosteroids. Annals of allergy, asthma & immunology : official publication of the American College of Allergy, Asthma, & Immunology. pp. 51-59.
170. Nathan RA, Li JT, Finn A, Jones R, Payne JE, et al. (2000) A dose-ranging study of fluticasone propionate administered once daily via multidose powder inhaler to patients with moderate asthma. Chest. pp. 296-302.
171. Nathan RA, Nayak AS, Graft DF, Lawrence M, Picone FJ, et al. (2001) Mometasone furoate: efficacy and safety in moderate asthma compared with beclomethasone dipropionate.

Annals of allergy, asthma & immunology : official publication of the American College of Allergy, Asthma, & Immunology. pp. 203-210.
172. Nathan RA, Bleecker ER, Kalberg C, Fluticasone Propionate Study G (2001) A comparison of short-term treatment with inhaled fluticasone propionate and zafirlukast for patients with persistent asthma. The American journal of medicine. pp. 195-202.
173. Nelson HS, Busse WW, Kerwin E, Church N, Emmett A, et al. (2000) Fluticasone propionate/salmeterol combination provides more effective asthma control than low-dose inhaled corticosteroid plus montelukast. The Journal of allergy and clinical immunology. pp. 1088-1095.
174. Nelson HS, Wolfe JD, Gross G, Greos LS, Baitinger L, et al. (2003) Efficacy and safety of fluticasone propionate 44 microg/salmeterol 21 microg administered in a hydrofluoroalkane metered-dose inhaler as an initial asthma maintenance treatment. Annals of allergy, asthma & immunology : official publication of the American College of Allergy, Asthma, & Immunology. pp. 263-269.
175. O'Connor B, Bonnaud G, Haahtela T, Luna JM, Querfurt H, et al. (2001) Dose-ranging study of mometasone furoate dry powder inhaler in the treatment of moderate persistent asthma using fluticasone propionate as an active comparator. Annals of allergy, asthma & immunology : official publication of the American College of Allergy, Asthma, & Immunology. pp. 397-404.
176. Pauwels RA, Pedersen S, Busse WW, Tan WC, Chen YZ, et al. (2003) Early intervention with budesonide in mild persistent asthma: a randomised, double-blind trial. Lancet. pp. 1071-1076.
177. Pearlman DS, Peden D, Condemi JJ, Weinstein S, White M, et al. (2004) Efficacy and safety of fluticasone propionate/salmeterol HFA 134A MDI in patients with mild-to-moderate persistent asthma. The Journal of asthma : official journal of the Association for the Care of Asthma. pp. 797-806.
178. Pearlman DS, Noonan MJ, Tashkin DP, Goldstein MF, Hamedani AG, et al. (1997) Comparative efficacy and safety of twice daily fluticasone propionate powder versus placebo in the treatment of moderate asthma. Annals of allergy, asthma & immunology : official publication of the American College of Allergy, Asthma, & Immunology. pp. 356-362.
179. Pearlman DS, Stricker W, Weinstein S, Gross G, Chervinsky P, et al. (1999) Inhaled salmeterol and fluticasone: a study comparing monotherapy and combination therapy in asthma. Annals of allergy, asthma & immunology : official publication of the American College of Allergy, Asthma, & Immunology. pp. 257-265.
180. Pinnas JL, Noonan MJ, Weinstein SF, Chervinsky P, Scott CA, et al. (2005) Fluticasone propionate HFA-134a pressurized metered-dose inhaler in adolescents and adults with moderate to severe asthma. The Journal of asthma : official journal of the Association for the Care of Asthma. pp. 865-871.
181. Rabe KF, Pizzichini E, Ställberg B, Romero S, Balanzat AM, et al. (2006) Budesonide/formoterol in a single inhaler for maintenance and relief in mild-to-moderate asthma: a randomized, double-blind trial. Chest. pp. 246-256.
182. Raphael GD, Lanier RQ, Baker J, Edwards L, Rickard K, et al. (1999) A comparison of multiple doses of fluticasone propionate and beclomethasone dipropionate in subjects with persistent asthma. The Journal of allergy and clinical immunology. pp. 796-803.
183. Scicchitano R, Aalbers R, Ukena D, Manjra A, Fouquert L, et al. (2004) Efficacy and safety of budesonide/formoterol single inhaler therapy versus a higher dose of budesonide in moderate to severe asthma. Current medical research and opinion. pp. 1403-1418.
184. Shapiro G, Lumry W, Wolfe J, Given J, White MV, et al. (2000) Combined salmeterol 50 microg and fluticasone propionate 250 microg in the diskus device for the treatment of asthma. American Journal of Respiratory and Critical Care Medicine. pp. 527-534.

185. Sheffer AL, LaForce C, Chervinsky P, Pearlman D, Schaberg A (1996) Fluticasone propionate aerosol: efficacy in patients with mild to moderate asthma. Fluticasone Propionate Asthma Study Group. The Journal of family practice. pp. 369-375.
186. Wardlaw A, Larivee P, Eller J, Cockcroft DW, Ghaly L, et al. (2004) Efficacy and safety of mometasone furoate dry powder inhaler vs fluticasone propionate metered-dose inhaler in asthma subjects previously using fluticasone propionate. Annals of allergy, asthma & immunology : official publication of the American College of Allergy, Asthma, & Immunology. pp. 49-55.
187. ZuWallack RL, Rosen JP, Cohen L, Rachelefsky GS, Gong H, et al. (1997) The effectiveness of once-daily dosing of inhaled flunisolide in maintaining asthma control. The Journal of allergy and clinical immunology. pp. 278-285.
188. ZuWallack R, Adelglass J, Clifford DP, Duke SP, Wire PD, et al. (2000) Long-term efficacy and safety of fluticasone propionate powder administered once or twice daily via inhaler to patients with moderate asthma. Chest. pp. 303-312.
